# Evaluation of Sedative and Hypnotic Activity of Ethanolic Extract of *Scoparia dulcis* Linn.

**DOI:** 10.1155/2015/873954

**Published:** 2015-03-12

**Authors:** Md. Moniruzzaman, Md. Atikur Rahman, Afia Ferdous

**Affiliations:** ^1^College of Pharmacy, Dongguk University, Goyang 410-820, Republic of Korea; ^2^Department of Pharmacy, Stamford University Bangladesh, 51 Siddeswari Road, Dhaka 1217, Bangladesh

## Abstract

*Scoparia dulcis* Linn. (SD) is a perennial herb that has been well studied for its antioxidant, anti-inflammatory, antidiabetic, and hepatoprotective effects. However, scientific information on SD regarding the neuropharmacological effect is limited. This study evaluated the sedative and hypnotic effect of the ethanolic extract of whole plants of *Scoparia dulcis* (EESD). For this purpose, the whole plants of *S. dulcis* were extracted with ethanol following maceration process and tested for the presence of phytochemical constituents. The sedative and hypnotic activity were then investigated using hole cross, open field, hole-board, rota-rod, and thiopental sodium-induced sleeping time determination tests in mice at the doses of 50, 100, and 200 mg/kg of EESD. Diazepam at the dose of 1 mg/kg was used as a reference drug in all the experiments. We found that EESD produced a significant dose-dependent inhibition of locomotor activity of mice both in hole cross and open field tests (*P* < 0.05). Besides, it also decreased rota-rod performances and the number of head dips in hole-board test. Furthermore, EESD significantly decreased the induction time to sleep and prolonged the duration of sleeping, induced by thiopental sodium. Taken together, our study suggests that EESD may possess sedative principles with potent hypnotic properties.

## 1. Introduction

Sedative and hypnotics are the drugs which can reduce anxiety and produce a calming effect by inducing the onset of sleep as well as maintaining sleeping duration [1]. Nowadays, these drugs are extensively used in treatment of different psychiatric disorders including anxiety and insomnia. However, continuous use of these currently available sedative-hypnotic therapies tends to have serious side effects ranging from respiratory, digestive, and immune system dysfunctions to deterioration of cognitive function, physical dependence, and tolerance [2]. Thus, development of new sedative-hypnotic drugs with fewer side effects has been suggested to be a promising approach to combat different psychiatric disorders.

*Scoparia dulcis *L. (family: Scrophulariaceae), commonly known as sweet broomweed, is a perennial herb widely distributed in tropical and subtropical regions. In these regions this plant has been used in folk medicine to treat a wide range of diseases mainly stomach troubles, hypertension, fever, diabetes, bronchitis, pain, and inflammation [3, 4]. Based on the traditional uses, researchers tried to explore and validate the scientific basis of therapeutic efficacies of this plant against different diseases conditions. Their findings provided substantial scientific evidence for the beneficial impact of this plant highlighting its antidiabetic, anti-inflammatory, and antioxidant capacity* in vivo *[3–5], impact on lipid peroxidation [6],* in vivo *antianemic properties [7], protective role on insulinoma cell line RINm5F [8] on kidney, heart, and liver of rats exposed to cadmium [5], and the antibacterial and antifungal properties [9]. Preliminary phytochemical screening revealed that this plant is rich in flavonoids and terpenes including scopadulcic acids A and B, scopadiol, scopadulciol, scopadulin, scoparic acids A–C, and betulinic acid. Additionally identified terpenoids of broomweed involve alpha-amyrin, friedelin, glutinol, and ifflaionic acid, where all of these phytochemicals were reported to show their biological activity against different pathological conditions [3, 10]. However, so far, there is no report demonstrating the sedative-hypnotic activity of* Scoparia dulcis*, which prompted us to design the present study to evaluate the role of this plant on the central nervous system (CNS) in mice. We found that the ethanolic extract of* Scoparia dulcis* (EESD) significantly reduced the locomotor activity and motor coordination of mice. Moreover, pretreatment with this extract also potentiates thiopental sodium-induced hypnosis in mice by decreasing onset of sleep and prolonging sleeping duration. Therefore, our findings strongly support the sedative and hypnotic activities of EESD and suggest that it can be useful to treat different psychiatric disorders including insomnia.

## 2. Materials and Methods

### 2.1. Plant Collection and Extraction

The whole plant of* Scoparia dulcis *was collected from Narsingdi, Bangladesh, in January 2012. The samples were then identified by Bushra Khan, Principal Scientific Officer, Bangladesh National Herbarium, Mirpur, Dhaka, Bangladesh. A voucher specimen (DACB: 36152) has been deposited in the Herbarium for further reference. Powdered dried plants (250 g) were macerated with 420 mL of ethanol with occasional stirring at 25 ± 2°C for 3 days. The extract was then filtered using a Buchner funnel and a sterilized cotton filter. The solvent was completely removed by rotary evaporator and 14 g extract (Yield 5.6%) was obtained. This crude extract was then used for further studies.

### 2.2. Animals

Swiss albino mice of 20–25 g were collected from Animal Resources Branch of the International Center for Diarrhoeal Disease Research, Bangladesh (ICDDR, B). The animals were kept in standard laboratory conditions (relative humidity 55–60%; room temperature 25 ± 2°C; 12 h light/dark cycle) and were provided with standard diet (ICDDR, B formulated) and clean water* ad libitum* during acclimatization period. The animals were acclimatized to the laboratory environment for a period of 14 days prior to performing the experiments. The animals were fasted overnight before the experiments. All the experimental animals were treated following the Ethical Principles and Guidelines for Scientific Experiments on Animals (1995) formulated by The Swiss Academy of Medical Sciences and the Swiss Academy of Sciences. All experimental protocols were approved by the Institutional Ethics Committee (SUB/IAEC/12.01).

### 2.3. Drugs and Treatments

The animals were divided into five groups containing 5–7 animals each for control, standard, and test sample for every experiment. EESD was dissolved in DMSO and orally administered to the test animals 30 min before the experiments at the doses of 50, 100, and 200 mg/kg body weight. On the other hand, the standard drug diazepam (1 mg/kg) employed in all tests was administered intraperitoneally (i.p.) 15 min before the experiments where thiopental sodium (20 mg/kg) in sleeping time measurement test was administered 15 min after treatment with diazepam and 30 min of vehicle (DMSO) or EESD. The animals in control group received vehicle orally at the dose of 0.1 mL/mouse 30 min before the experiments.

### 2.4. Phytochemical Screening

The ethanolic extract of* Scoparia dulcis *was qualitatively investigated for the presence of alkaloids, flavonoids, tannins, saponins, and gum according to the standard procedures [11].

### 2.5. Acute Toxicity Test

The mice were divided into control and three test groups each containing five animals. EESD was administered to the animals orally at doses of 1000, 2000, and 3000 mg/kg. The mice were given access to food and water* ad libitum* and all animals were observed for allergic symptoms and mortality for the next 72 h [12].

### 2.6. Hole Cross Test

For this experiment, a cage was used having a size of 30 × 20 × 14 cm with a fixed partition in the middle having a hole of 3 cm diameter [13]. The animals were treated with either vehicle or drug or EESD and allowed to cross the hole from one chamber to another. Mice were observer for 3 min and the number of passages was recorded at 30, 60, 90, and 120 min following the treatments.

### 2.7. Open Field Test

This method was carried out as described by Gupta et al. [14]. The open field apparatus consisted of a wooden field of half square meter with a series of squares alternatively painted in black and white. It had a wall of 50 cm height and was placed in a dimly lit room. Mice were treated with vehicle, extract, or diazepam and were placed in the middle of the open field. Then the number of squares visited by the animals was counted for 3 minutes at 30, 60, 90, and 120 min after the treatments.

### 2.8. Hole-Board Test

The hole-board test was performed according to the previously described method by Ozturk et al. [15], with slight modifications. For this test, we used a flat platform of 60 cm × 30 cm in diameter with 16 evenly spaced holes. In brief, thirty minutes after vehicle or EESD and 15 min after diazepam administration, each animal was allowed to move on the platform and the number of head dips into the holes was counted for 5 min.

### 2.9. Test for Motor Coordination (Rota-Rod Test) in Mice

This test was performed using a horizontal rotation rod (Ugo Basile, Varese, Italy) set at a rate of 20 revolutions per min. Mice that were able to remain on the rod longer than 180 s were selected and divided into desired groups. Thirty minutes after the vehicle or EESD administration, each mouse was placed on the rod for 180 s where diazepam was given 15 min before the experiment [16]. Then the falling time from the rotating rod was recorded for each mouse.

### 2.10. Thiopental Sodium-Induced Sleeping Time Determination

Thiopental sodium-induced sleeping time was evaluated according to the previously described method [17]. Thirty min after vehicle or EESD and 15 min after diazepam treatment, thiopental sodium (TS) was administered to each animal intraperitoneally at the dose of 20 mg/kg. Then the animals were observed for the time to lose their righting reflex, immediately after thiopental sodium injection (latent period) and the duration of sleep (time between the loss and recovery of reflex) induced by TS.

### 2.11. Statistical Analysis

The results are presented as Mean ± SEM. The statistical analysis was performed using one-way analysis of variance (ANOVA) followed by Dunnett's post hoc test, except for the hole cross test and open field test. For these tests, two-way ANOVA followed by Bonferroni's post hoc tests was adopted. All statistical analysis was performed using SPSS 11.5 software.

## 3. Results and Discussions

The present study investigated the putative CNS effect of an ethanolic extract of whole plants of* S. dulcis*. The results showed that EESD exerts sedative and hypnotic effect on the CNS. Moreover, it was also found that acute oral administration of EESD at the doses of 1000, 2000, and 3000 mg/kg did not produce any allergic manifestations or mortality during the observation period of 72 h after administration. Therefore, it is conceivable that EESD may not be toxic at our experimental doses up to 3000 mg/kg.

We started our investigation for the sedative effects of EESD by recording spontaneous locomotor activity of mice in hole cross and open field tests. In these tests, any agents with sedative properties will produce a decrease in the number of movements, interpreted as a decrease in curiosity of the new environment [13, 18]. Our result demonstrated that the oral administration of EESD in all doses (50, 100, and 200 mg/kg) caused a marked reduction in number of hole crossed (Table 1). The suppressive effect was found at 30 min and continued up to 120 min after administration of EESD. Also, similar types of responses were observed in the open field test. EESD at all tested doses produced significant (*P* < 0.05) inhibition of locomotion that was maintained from 30 min to 120 min of observation period (Table 2). This ability of EESD to suppress the locomotor activity suggests that the extract is endowed with central nervous system depressant activity.

Another important observation was achieved in the hole-board test. This test is well-established as a means to assay potential anxiolytic and sedative effects of any agents by observing the exploratory behavior in rodents. This experiment is advantageous due to its methodological simplicity and several behavioral responses of an animal can be readily observed and quantified when exposed to an unfamiliar environment. It was found that the head-dipping behavior of the animals is directly related to their emotional state [19]. Based on this observation, it was suggested that the expression of an anxiolytic state in animals might be reflected by an increase in head-dipping behavior [19], while a decrease in the number of head dips was found to be correlated with the depressant effect [20, 21]. Our results revealed that the ethanolic extract of* S. dulcis* caused a dose-dependent reduction in head-dip response in the animals (23.84, 45.55, and 70.46% head-dip inhibition for 50, 100, and 200 mg/kg doses, resp.) suggesting that the extract possesses sedative activity rather than anxiolytic potentials. The observed effects in the treated groups were significantly different (*P* < 0.05) from that of the control group (Table 3).

The rota-rod test is a widely used method to evaluate the motor coordination or muscle relaxant effect in rodents. Our results demonstrated that treatment with EESD at 50, 100, and 200 mg/kg doses markedly reduced the falling latency of the animals from the rotating rod. The decrement of the latency was calculated as 12.88, 43.98, and 66.05% of the respective control, for 50, 100, and 200 mg/kg doses, respectively, where the effect produced by the latter two doses are statistically significant (*P* < 0.05) (Table 4). It is well established that some benzodiazepines like diazepam cause muscle weakness [22], decrease of ambulatory activity, and sedation, thus impairing the performance of rodents in the rota-rod [23, 24]. As expected, we also found that diazepam at 1 mg/kg dose caused muscle relaxation of the animals causing a decrement of the falling time in rota-rod. This same fashion of effect, produced by EESD and diazepam, influenced us to conceive that EESD can induce sedative effect by affecting the general activity and motor coordination of the animals.

Our above findings were further supported by the results observed in thiopental sodium-induced sleeping time determination test. This test is a classical method in behavioral pharmacology to investigate the sedative and hypnotic properties. In our study, the acute oral treatment with 50, 100, and 200 mg/kg of EESD 30 min before the TS injection significantly modified the latency to induce sleep as well as increasing duration of hypnosis induced by TS, as depicted in Table 5. As expected, similar types of effects were observed by the administration with diazepam at 1 mg/kg dose. Substantial evidence revealed that the CNS depressant barbiturates, such as TS, bind to the barbiturate binding site on the GABA_A_ receptor complex and potentiate GABA-mediated hyperpolarization of postsynaptic neurons [25]. Our results suggest that there might be a relationship between the sedative effect produced by EESD and the sedation inductive capacity of diazepam. Therefore, it is possible that the GABAergic system may participate in the EESD-induced enhancement of the effects of TS.

Our preliminary phytochemical screening confirmed the presence of alkaloids, glycosides, flavonoids, carbohydrates, saponins, steroids, and tannins in the ethanolic extract of* S. dulcis* (Table 6). There are several reports which demonstrated that the alkaloids, glycosides, and flavonoids rich plant and plant extracts possess sedative, anxiolytic, and antiepileptic properties mediated through their affinity (*in vitro*) with benzodiazepine site of GABAergic complex system or are direct or indirect modulators of this receptor [25–29]. Besides, nonspecific CNS depression can also be attributed by tannin [30]. Therefore it appears that the abovementioned phytochemicals present in the EESD may contribute at least in part to the sedative and hypnotic effects on the CNS.

## 4. Conclusion

In conclusion, the present findings in our study indicate that* S. dulcis* possesses strong sedative and hypnotic activities. The effect is rapid, long-lasting, and statistically significant at all the experimental doses tested. However, further studies are needed to isolate bioactive compound(s) and elucidate the precise molecular mechanisms responsible for the pharmacological activities of the plant.

## Figures and Tables

**Table 1:** Effect of EESD on hole cross test.

Treatment	Dose (mg/kg)	Number of holes crossed (% of inhibition)				
		Pretreatment	30 min	60 min	90 min	120 min
Control	0.1 mL/mouse	19 ± 1.58	17.60 ± 0.75	15.6 ± 0.93	13.4 ± 0.51	12.4 ± 0.51
Diazepam	1	21.2 ± 1.63	5.2 ± 1.16^*^ (70.45)	3.2 ± 0.37^*^ (79.49)	2.0 ± 0.45^*^ (85.07)	2.2 ± 0.74^*^ (82.26)
EESD	50	18.6 ± 1.69	12.6 ± 1.54^*^ (28.41)	10.2 ± 1.20^*^ (34.62)	7.2 ± 1.16^*^ (46.27)	4.2 ± 0.97^*^ (66.13)
EESD	100	20.4 ± 1.08	8.80 ± 0.49^*^ (50.00)	7.8 ± 1.02^*^ (50.00)	3.6 ± 0.81^*^ (73.13)	3.2 ± 0.86^*^ (74.19)
EESD	200	21 ± 0.84	6.0 ± 0.84^*^ (65.91)	2.0 ± 0.71^*^ (87.18)	1.6 ± 0.4^*^ (88.06)	1.2 ± 0.37^*^ (90.32)

Values are presented as the Mean ± SEM (*n* = 5). EESD = ethanolic extract of *Scoparia dulcis*; ^*^ *P* < 0.05 compared with the control group (two-way ANOVA followed by Bonferroni's test).

**Table 2:** Effect of EESD on open field test.

Treatment	Dose (mg/kg)	Number of squares crossed (% of inhibition)				
		Pretreatment	30 min	60 min	90 min	120 min
Control	0.1 mL/mouse	102.8 ± 2.99	93.2 ± 3.31	83 ± 3.67	67.8 ± 1.93	56.4 ± 2.79
Diazepam	1	97.4 ± 6.08	47.4 ± 1.66^*^ (49.14)	29.2 ± 2.46^*^ (64.82)	13.6 ± 2.09^*^ (79.94)	8.8 ± 0.86^*^ (84.40)
EESD	50	95.2 ± 5.83	59.4 ± 1.66^*^ (36.27)	43.4 ± 3.50^*^ (47.71)	27.6 ± 5.85^*^ (59.29)	19.6 ± 2.32^*^ (65.25)
EESD	100	99.4 ± 6.40	46.6 ± 9.32^*^ (50)	31.8 ± 4.87^*^ (61.69)	15.8 ± 2.91^*^ (76.70)	9.8 ± 2.76^*^ (82.62)
EESD	200	98.4 ± 5.34	39.6 ± 1.50^*^ (57.51)	20.6 ± 2.25^*^ (75.18)	7.6 ± 0.93^*^ (88.79)	3.20 ± 0.86^*^ (94.33)

Values are presented as the Mean ± SEM (*n* = 5). EESD = ethanolic extract of *Scoparia dulcis*; ^*^ *P* < 0.05 compared with the control group (two-way ANOVA followed by Bonferroni's test).

**Table 3:** Effect of EESD on hole-board test.

Treatment	Dose (mg/kg)	Responses	
		Number of head dips	% inhibition
Control	0.1 mL/mouse	56.2 ± 2.27	0
Diazepam	1	13.8 ± 2.04^*^	75.44
EESD	50	42.8 ± 2.42^*^	23.84
EESD	100	30.6 ± 2.71^*^	45.55
EESD	200	16.6 ± 1.47^*^	70.46

Values are presented as the Mean ± SEM (*n* = 5). EESD = ethanolic extract of *Scoparia dulcis*; ^*^ *P* < 0.05 compared with the control group (one-way ANOVA followed by Dunnett's test).

**Table 4:** Effect of EESD on motor coordination of mice.

Treatment	Dose (mg/kg)	Responses	
		Rota-rod performance (s)	% inhibition
Control	0.1 mL/mouse	119.6 ± 8.47	0
Diazepam	1	26.6 ± 2.99^*^	77.76
EESD	50	104.2 ± 4.26	12.88
EESD	100	67.0 ± 3.61^*^	43.98
EESD	200	40.6 ± 4.82^*^	66.05

Values are presented as the Mean ± SEM (*n* = 5). EESD = ethanolic extract of *Scoparia dulcis*; ^*^ *P* < 0.05 compared with the control group (one-way ANOVA followed by Dunnett's test).

**Table 5:** Effect of EESD on thiopental sodium-induced sleeping time.

Treatment	Dose (mg/kg)	Responses	
		Onset of sleeping	Sleeping duration
Control	0.1 mL/mouse	7.81 ± 0.47	88.80 ± 4.91
Diazepam	1	5.74 ± 0.26^*^	177.60 ± 2.44^*^
EESD	50	7.18 ± 0.23	107.60 ± 8.08
EESD	100	6.41 ± 0.37^*^	130.40 ± 7.69^*^
EESD	200	5.93 ± 0.27^*^	145.20 ± 6.76^*^

Values are presented as the Mean ± SEM (*n* = 5). EESD = ethanolic extract of *Scoparia dulcis*; ^*^ *P* < 0.05 compared with the control group (one-way ANOVA followed by Dunnett's test).

**Table 6:** Phytochemicals identified in EESD.

Phytochemicals	Name of the tests	Observed changes	Result
Alkaloids	Mayer's test	Yellowish buff color precipitate	+
	Hager's test	Yellow crystalline precipitate	−
	Wagner's test	Brown or deep brown precipitate	−
	Dragendorff's test	Orange or orange-brown precipitate	+
	Tannic acid test	Buff color precipitate	−

Glycosides	General test	Yellow color	+
	Test for glucoside	Production of brick-red precipitation	+

Flavonoids	Hydrochloric acid reduction test	Red color	+

Carbohydrates	Molisch's test	A red or reddish violet ring is formed at the junction of two layers and on shaking a dark purple solution is formed	+
	Barfoed's test (general test for monosaccharides)	Red precipitate	−
	Fehling's test	A red or brick-red precipitate	+
	Benedict's test		−
	Test for reducing sugar	A brick-red precipitate	+

Saponins	Frothing test	Formation of stable foam	+

Steroids	Sulphuric acid test	Red color	+

Tannins	Ferric chloride test	Blue green color	−
	Alkaline reagent test	Yellow to red precipitate	+
